# Case-control study on post-COVID-19 conditions reveals severe acute infection and chronic pulmonary disease as potential risk factors

**DOI:** 10.1016/j.isci.2024.110406

**Published:** 2024-06-28

**Authors:** Pritha Ghosh, Michiel J.M. Niesen, Colin Pawlowski, Hari Bandi, Unice Yoo, Patrick J. Lenehan, Praveen Kumar-M, Mihika Nadig, Jason Ross, Sankar Ardhanari, John C. O’Horo, A.J. Venkatakrishnan, Clifford J. Rosen, Amalio Telenti, Ryan T. Hurt, Venky Soundararajan

**Affiliations:** 1 nference Labs, Bengaluru, India; 2 nference, Inc., Cambridge, MA 02139, USA; 3 nference, Inc., 18 3rd St. S.W., Rochester, MN 55902, USA; 4 nference, Inc., 2424 Erwin Road, Durham, NC 27705, USA; 5 Mayo Clinic, Rochester, MN, USA; 6 Maine Medical Center, Portland, ME 04102, USA; 7 RECOVER Maine, MaineHealth Institute for Research, Scarborough, ME, USA; 8 Vir Biotechnology, Inc., San Francisco, CA, USA; 9 Anumana, Inc., Cambridge, MA 02139, USA

**Keywords:** Immunology, Respiratory medicine, Public health

## Abstract

Post-COVID-19 conditions (long COVID) has impacted many individuals, yet risk factors for this condition are poorly understood. This retrospective analysis of 88,943 COVID-19 patients at a multi-state US health system compares phenotypes, laboratory tests, medication orders, and outcomes for 1,086 long-COVID patients and their matched controls. We found that history of chronic pulmonary disease (CPD) (odds ratio: 1.9, 95% CI: [1.5, 2.6]), migraine (OR: 2.2, [1.6, 3.1]), and fibromyalgia (OR: 2.3, [1.3, 3.8]) were more common for long-COVID patients. During the acute infection phase long COVID patients exhibited high triglycerides, low HDL cholesterol, and a high neutrophil-lymphocyte ratio; and were more likely hospitalized (5% vs. 1%). Our findings suggest severity of acute infection and history of CPD, migraine, chronic fatigue syndrome (CFS), or fibromyalgia as risk factors for long COVID. These results suggest that suppressing acute disease severity proactively, especially in patients at high risk, can reduce incidence of long COVID.

## Highlights

• Compared EHR data for 1,086 patients with/without post-COVID conditions
• Post-COVID conditions linked to chronic pulmonary disease, migraine, and fibromyalgia
• Post-COVID conditions linked to elevated inflammation markers
• Patients with post-COVID conditions showed more severe acute COVID symptoms

## Graphical abstract

**Figure:**
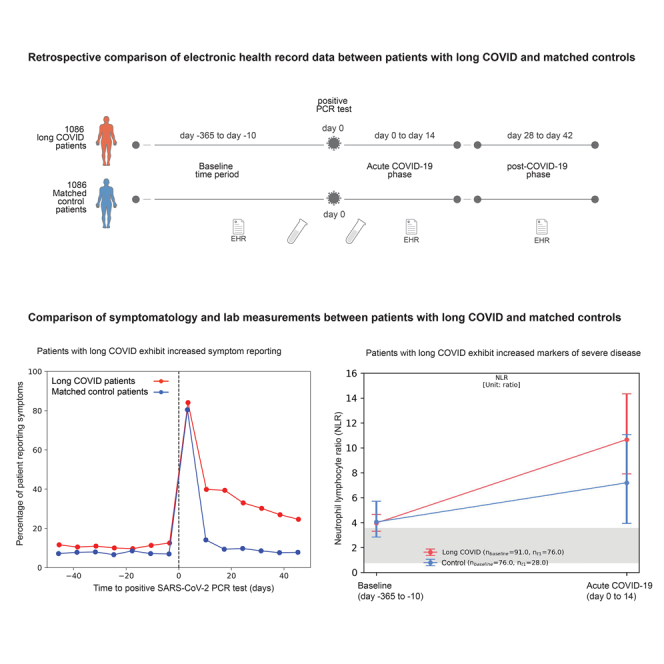

## Introduction

According to CDC estimates, approximately 58% of the United States population has had a SARS-CoV-2 infection at least once through February 2022,^1^ and the total number of confirmed COVID-19 deaths surpassed 1 million in May 2022.^2^ Given the high prevalence of COVID-19 and its large burden on health systems and society overall, it is a public health imperative to understand the short-, medium-, and long-term effects of this disease so that optimal care can be offered to COVID-19 patients during their infection and their convalescence. There is mounting evidence that SARS-CoV-2 infection may have significant long-term health effects for some individuals. For example, some individuals, particularly those infected with earlier variants of SARS-CoV-2, may experience persistent loss of taste and/or smell.^3^ The WHO developed a clinical case definition for post-COVID-19 conditions (also known as “long COVID”), which include fatigue, shortness of breath, and cognitive dysfunction as common symptoms, and persist or develop 3 months after the initial COVID-19 diagnosis, and last for at least two months after infection.^4^ In October 2021, an ICD code for long COVID was adopted internationally (U09.9). According to the National Center for Health Statistics (NCHS) Household Pulse Survey, approximately 34% of individuals who were infected with COVID-19 report symptoms lasting three months or more after their infection.^5^ One large retrospective study found that anosmia, hair loss, sneezing, ejaculation difficulty, and reduced libido were relatively presented more among patients with COVID-19 in the longer term compared to those patients without COVID-19, and risk factors include female sex, belonging to an ethnic minority, socioeconomic deprivation, smoking, obesity, and a wide range of comorbidities.^6^ Currently, prospective studies are underway to characterize the long-term sequelae of COVID-19, including the CDC INSPIRE study,^7^ and the NIH RECOVER initiative.^8^

Here, we conduct a large-scale retrospective analysis of de-identified electronic health records from a multi-state health system to characterize long COVID conditions and associated risk factors. We consider a study population of patients with long COVID based on an ICD code diagnosis and control patients with COVID-19 and without long COVID diagnosis. We perform 1:1 matching to ensure that the long-COVID and control groups are balanced on clinical characteristics, including demographics, date of infection, geography, and the number of prior laboratory testing encounters. We examined trends in lab test measurements for these two matched cohorts during a baseline phase before COVID-19 diagnosis and an acute COVID-19 phase. In addition, we compared other clinical features between these two cohorts including hospitalization, diagnoses, medications, and signs and symptoms captured in clinical notes.

## Results

The study population included 88,943 patients with a positive PCR test for SARS-CoV-2, including 1,140 patients with an ICD-10 code diagnosis for long COVID (U09.9). In Table S1, we provide the clinical characteristics of the unmatched cohorts. We observed that the observed rate of long COVID was higher among females compared to males (odds ratio: 1.42, 95% CI: [1.26, 1.60]). In addition, the median age of individuals in the long COVID cohort was significantly higher compared to the control patients (Table S1). We performed a matched analysis to control for differences in demographics and other potential confounding factors for long COVID diagnosis (see STAR Methods section for details). In Table 1, we provide the comorbidities and clinical outcomes for the final 1:1 matched long COVID and control groups, which each included 1,086 patients. In Table S4, we provide a summary of the matched clinical characteristics for these cohorts. For the rest of this section, we present the results based on these matched cohorts.

**Table 1:** Comorbidities and clinical outcomes of long COVID and matched control groups

	Long COVID cohort (matched)	Control patients (matched)	Odds Ratio [95% CI]
Number of individuals	1,086	1,086	–
Fully vaccinated before infection^a^ (%)			
Pfizer (two or more doses)	37	41	0.85 [0.71, 1.00]
Moderna (two or more doses)	16	20	0.78 [0.62, 0.97]∗
Janssen (one or more doses)	5	4	1.37 [0.90, 2.07]
Any other vaccine (two or more doses)	0	<1	–
Charlson comorbidities in baseline phase (%)			
Cancer	6	6	0.98 [0.68, 1.41]
Cerebrovascular disease	3	3	0.94 [0.58, 1.53]
Chronic pulmonary disease	15	8	1.94 [1.48, 2.55]∗∗∗
Congestive heart failure	8	6	1.40 [1.00, 1.97]
Dementia	<1	<1	–
Diabetes without chronic complication	12	10	1.14 [0.87, 1.50]
Hemiplegia or paraplegia	<1	<1	0.33 [0.07, 1.65]
Metastatic solid tumor	1	2	0.63 [0.34, 1.20]
Mild liver disease	3	5	0.69 [0.45, 1.06]
Moderate or severe liver disease	<1	<1	–
Myocardial infarction	3	1	1.83 [0.99, 3.40]
Peptic ulcer disease	1	<1	2.52 [0.97, 6.52]
Peripheral vascular disease	7	5	1.31 [0.92, 1.86]
Renal disease	14	10	1.40 [1.08, 1.82]∗
Rheumatic disease	5	3	1.48 [0.97, 2.27]
at least one of the listed comorbidities	40	36	1.21 [1.01, 1.44]∗
Auto-immune diseases and potentially related conditions in baseline phase (%)			
Chronic Fatigue Syndrome	1	<1	2.16 [0.88, 5.32]
Postural Tachycardia Syndrome Without Hypotension	<1	<1	–
Fibromyalgia	4	2	2.25 [1.32, 3.84]∗
Migraine	10	5	2.22 [1.57, 3.14]∗∗∗
at least one of the listed conditions	13	6	2.27 [1.67, 3.08]∗∗∗
Individuals admitted 0–14 days post-infection (%)			
Hospitalized	5	1	4.74 [2.58, 8.70]∗∗∗
ICU admission	3	1	2.63 [1.34, 5.15]∗
Intubated	3	1	2.29 [1.26, 4.16]∗

For each categorical variable, the percentage of patients in each cohort is shown along with the odds ratio and corresponding 95% confidence interval. Odds ratios that are statistically significant (*p*-value <0.05) are indicated with ∗, and those that are highly significant (*p*-value <0.001) are indicated with ∗∗∗. Odds ratios for comparisons with <1% of patients in both cohorts are not shown. The matched clinical characteristics for these two cohorts are provided in Table S4.

a Patients who have received COVID-19 vaccine doses from multiple manufacturers are also included here.

### Cough, difficulty breathing, and tiredness are the most commonly reported conditions for the long COVID cohort in the post-COVID-19 infection phase

**Figure 1:**
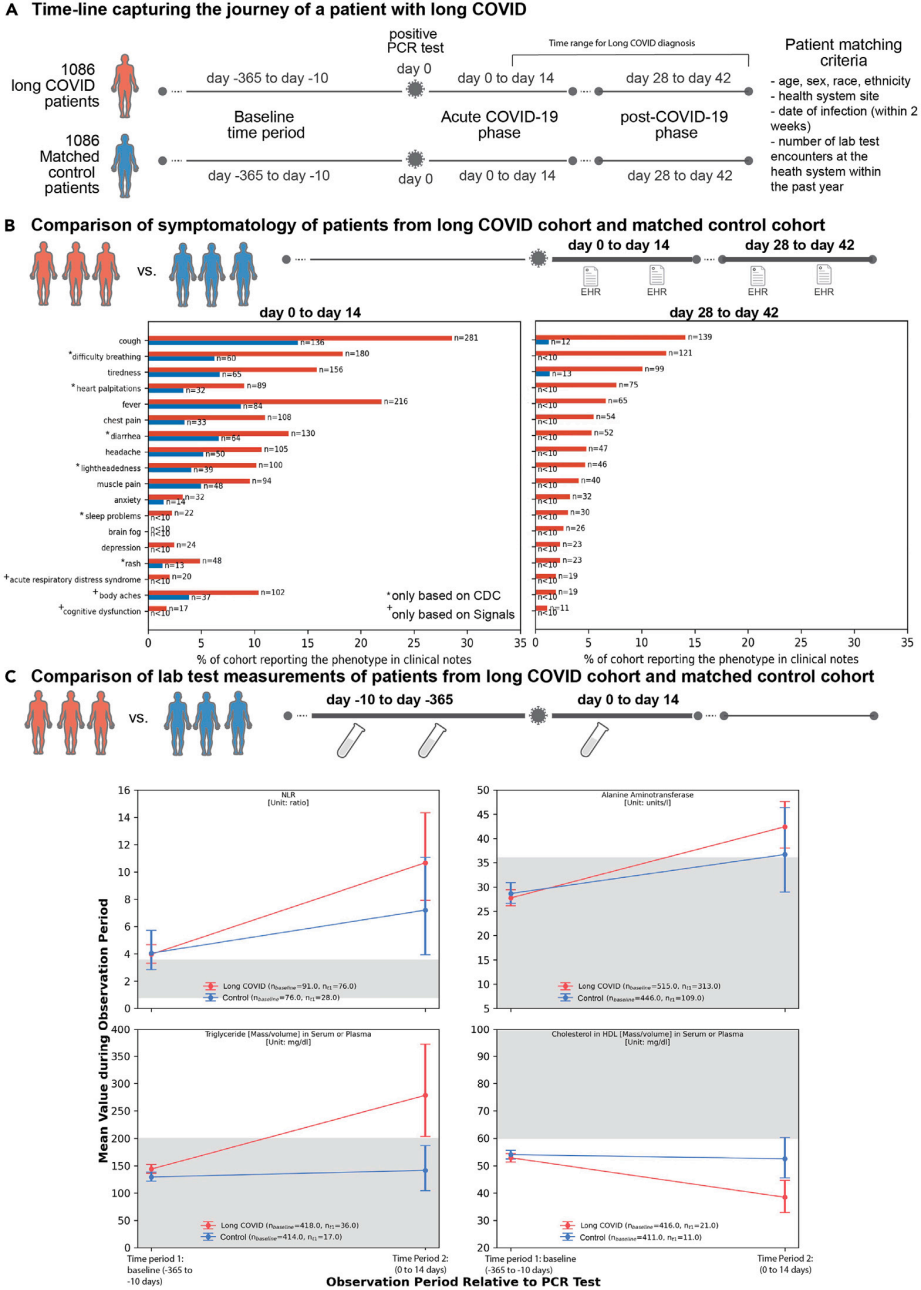
Study Overview (A) Timeline capturing the journey of a patient with long COVID. There are three main phases — (i) baseline (10–365 days before infection), (ii) acute COVID-19 (0–14 days after infection), and (iii) post-COVID-19 (28–42 days after infection). (B) Comparison of new onset symptoms and diseases recorded in EHR notes following a positive SARS-CoV-2 PCR test. Only phenotypes for which there is a significant difference in reporting (Fisher’s exact test, *p*-value <0.05) between the long COVID and control groups are shown. (C) Lab test with significant difference between the matched long COVID vs. control groups. For each lab test, mean test values and 95% confidence intervals are shown. The normal ranges for these lab tests^9^^,^^10^^,^^11^^,^^12^ are shaded in gray.

Next, we compared the rates of phenotypes reported in the clinical notes for the long COVID and control groups. For each phenotype, we observed higher rates in the long COVID patients compared to the control patients during both the acute COVID-19 and post-COVID-19 phases (Figures 1B and S1). Overall phenotype reporting was highest immediately following incidence of COVID-19, and for the long COVID cohort we found that phenotype reporting was increased compared to baseline reporting throughout (Figure S1). In contrast, phenotype reporting in the control patients was back at baseline levels within 20 days of their positive PCR test (Figure S1). For the long COVID cohort, almost all of the phenotypes were reported at lower rates during the post-COVID-19 phase compared to the acute COVID-19 phase, with the exception of brain fog (increase from <1% to 3%) and sleep problems (increase from 2% to 3%), which were both higher during the post-COVID-19 phase. The most common phenotypes in the long COVID cohort during the post-COVID-19 phase were cough (14%), difficulty breathing (12%), and tiredness (10%).

### Comparison of patient characteristics before infection

To identify features associated with a higher risk of developing post-COVID-19 conditions, we assessed differences during the baseline phase. We observed that patients with chronic lung disease had higher rates of long COVID diagnosis (odds ratio: 1.94, 95% CI: [1.48, 2.55]) (Table 1). This subpopulation of patients with chronic lung disease included patients with asthma, COPD, emphysema, and bronchiectasis (Figure S2). We also observed that individuals with renal disease had higher rates of long COVID diagnosis (odds ratio: 1.40, 95% CI: [1.08, 1.82]). Auto-immune diseases and conditions including migraine (odds ratio: 2.40, 95% CI: [1.77, 3.25]) and fibromyalgia (odds ratio: 2.25, 95% CI: [1.32, 3.84]) were also more common as pre-existing conditions in the long COVID cohort.

### Comparison of lab test measurements during acute infection

To determine whether there are clinical signatures of acute COVID-19 disease indicative of increased risk for subsequent post-COVID-19 conditions, we assessed differences during the acute COVID-19 phase. We observed differences consistent with increased acute disease severity in the long COVID cohort compared with their matched controls. In the long COVID cohort, hospital admission rates (within 14 days of infection) were significantly increased (Table 1, rate_longCOVID_: 5% vs. rate_control_: 1%, *p*-value: <0.001, odds ratio: 4.74 [2.58, 8.70]). Similarly, ICU admission rates were also significantly higher in the long COVID cohort (rate_longCOVID_: 3% vs. rate_control_: 1%, *p*-value: <0.01, odds ratio: 2.63 [1.34, 5.15])).

To assess whether laboratory measurements could predict onset of long COVID, we analyzed measurements for 82 tests contributed by more than ten patients in both the long COVID and the control patients during acute SARS-CoV-2 infection (Table S5). For 15 lab tests, the long COVID cohort exhibited a significant difference in mean test results (*p*-value <0.05) during the acute phase, both compared to the control patients during the acute phase, and the long COVID cohort during the baseline phase. Further, we compared the test results for these 15 lab tests to their known normal ranges (shaded region, Figures 1C, S3, and S4) 6 out of these 15 tests in the long COVID cohort exhibited mean test results outside the normal range in the acute phase (Figure 1C). Specifically, we observed increased levels of: neutrophil-lymphocyte ratio (mean_longCOVID_: 10.7, 95% CI: [7.9, 14.3] vs. mean_control_: 7.2 [3.9, 11.0]), alanine aminotransferase (42.4 [38.0, 47.6] u/L vs. 36.7 [28.9, 46.4] u/L), and serum triglyceride (278.5 [203.5, 372.7] mg/dL vs. 141.4 [104.3, 187.0] mg/dL). We also observed decreased levels of serum HDL cholesterol (38.4 [32.9, 44.6] mg/dL vs. 52.5 [45.5, 60.3] mg/dL).

### Comparison of ordered and administered medications during acute and post-acute infection

Concordant signals of more severe acute disease in long-COVID patients are also found when looking at medications ordered and administered during both the acute and post-acute phases (Figure S5; Table S6). Notably, antivirals, anticoagulants, and steroids were administered at significantly higher rates in the long COVID cohort. We did not observe a significant difference for monoclonal antibodies and administration of Albuterol was already elevated during the baseline, consistent with a higher prevalence of CPD in the long COVID cohort (Figure S6; Table S6).

## Discussion

In this study, we provide an in-depth characterization of a cohort of 1,086 patients diagnosed with long COVID compared with their matched controls. We found that the long COVID cohort was significantly enriched in patients with a history of CPD, fibromyalgia, and migraine. Additionally, we found that the patients that developed long COVID showed signs of more severe COVID-19 during their acute infection (0–14 days after infection) based on hospitalization, lab measurements, and medications administered.

Our findings are consistent with previous studies that have investigated long COVID signs and symptoms. We found that the most common phenotypes reported by long-COVID patients included cough, breathing difficulties, tiredness, and heart palpitations.^13^^,^^14^ We also found that long COVID patients exhibited low HDL cholesterol and high triglycerides levels in their serum during the acute COVID-19 phase, consistent with a previous retrospective study of 1,411 hospitalized COVID-19 patients.^15^ Previous studies have also shown that low albumin levels and elevated transaminases (ALT, AST) are associated with severe COVID-19 outcomes, and we observed the same to a lesser extent (Figure S3).^16^^,^^17^^,^^18^ Several of the long COVID characteristics observed in this study were also shown to be important variables for a recently described predictor of long COVID, including: difficulty breathing, dyspnea, cough, hospitalization, albuterol use, and CPD.^14^

In addition, both patients in the long COVID cohort and those in the control group had elevated levels of serum glucose level during the acute COVID-19 phase. In prior work, cases of metabolic dysfunction during and after SARS-CoV-2 infection have been reported ranging from new-onset diabetes mellitus (both Type I and Type 2) to asymptomatic insulin resistance and glucose intolerance.^19^^,^^20^^,^^21^^,^^22^ Acute SARS-CoV-2 infection can lead to metabolic dysfunction, including abnormal lipid profiles and sustained elevation in plasma glucose, through the chronic elaboration of cytokines, glucocorticoid treatment, and sustained stress related to severe infection and co-morbidity.^15^^,^^19^^,^^23^ There is evidence that these metabolic disturbances may be related to viral persistence in adipose tissue.^24^ Data from the current study also point to the potential for a metabolic signature. For example, changes in glucose disposal and lipid handling during the acute illness phase may be an early signal of further symptomatology and long COVID. Additionally, we found that NLR and HDL-C measurements are outside the normal range in control patients. Since these patients had an acute COVID-19 infection at the time their lab measurement was taken, this could explain their abnormal measurements.

There are several limitations for this analysis. First, this is a retrospective study carried out in a single multi-state health system, so the clinical characteristics of the study population are not representative of the entire population of patients with post-COVID-19 conditions. Given the retrospective nature of our analysis, there is a possibility of missed diagnoses of post-COVID conditions, which could potentially lead to an underestimation of the true prevalence of post-COVID conditions in the study population. Addressing this limitation, we acknowledge the potential impact on the study findings and the need for potential future prospective studies, which can help overcome this limitation. Second, the ICD-10 code for post-COVID-19 conditions only became available in the United States on October 1, 2021,^25^ so this analysis was restricted to long COVID cases reported during the Delta and Omicron waves of the pandemic. Third, although we control for demographics, time and date of infection, and the number of prior lab tests, additional confounding factors might explain the differences in the long COVID and control groups. For example, individuals in the long COVID cohort may engage in more health-seeking behaviors and thus have higher rates of reported comorbidities than the control group. Additionally, since patients in the long COVID cohort were more likely hospitalized, their EHR data may be more complete. This may partially explain the observed higher rates of medications or disease symptoms in the long COVID cohort due to improved recording. We therefore cannot draw causal relationships between observed enrichments and long COVID incidence. Fourth, not all of the patients in the long COVID and control groups underwent laboratory testing, so the observed distributions of lab values may not represent the distribution of lab values in the overall cohort. For example, some lab tests are ordered only in cases of a suspected diagnosis. In follow-up studies, methods for imputing missing values may be applied such as zero imputation, mean imputation, and multiple imputation.^26^ Fourth, our choice in study start date: June 1, 2021 poses another limitation. The reason for selecting this date was to capture individuals who were diagnosed with post-COVID conditions resulting from their index infection in June 2021. However, it is important to note that there might be a delay in the adoption and regular use of the ICD codes and for these conditions. This delay could lead to the possibility of missing individuals who may have been diagnosed with post-COVID conditions before June 2021 but were not included in the study due to the time lag in code adoption.

Overall, this study provides further clarity to the identification of risk factors for long COVID and motivates future research on the relationship between early interventions in COVID and the onset of long COVID. Future studies are needed to define individuals at highest risk for persistent symptomatology and possible interventions to forestall or prevent long COVID.

## STAR★Methods

### Key resources table

**Table:** 

REAGENT or RESOURCE	SOURCE	IDENTIFIER
**Software and algorithms**		

De-identfied electronic health record data	nference nSights	v4.005
List of long-COVID phenotypes from literature	nferX Signals (https://research.nferx.com/dv/202011/signals/)	Accessed August 18, 2022
Python	Python	v3.9.6
Software for statistical analysis	Numpy module for python	v1.23.3
Software for statistical analysis	Scipy module for python	v1.9.1
Software for statistical analysis	Statsmodels module for python	v0.13.2

### Resource availability

#### Lead contact

Further information and requests for resources and data should be directed to the lead contact, Dr. Venky Soundararajan (venky@nference.net).

#### Materials availability

No materials were generated as part of this study.

#### Data and code availability

• No new data were generated as part of this study. Data analyzed for this study are accessible via the nSights platform and can be made available to others upon reasonable requests to the lead contact, if access is granted. A proposal with a detailed description of the study objectives and the statistical analysis plan will be needed for evaluation of the reasonability of requests.
• This paper does not report original code.
• Any additional information required to reanalyze the data reported in this paper is available from the lead contact upon request.

#### nference platform with de-identified electronic health record data

We used the nference Clinical nSights platform to conduct this analysis. This platform includes de-identified records from over 6.9 million patients, spanning multiple US states. This de-identified environment includes structured tables derived from electronic health records (EHR) data such as ECG waveforms, diagnosis codes, laboratory tests, vital signs, medications administered, medications ordered, procedures, and flowsheets. In addition, this environment includes unstructured tables derived from the EHR, such as ECG, radiology and pathology reports, and clinical notes. All personally identifiable information in this environment (e.g., names, locations, dates) have been excluded or substituted using a best-in-class de-identification methodology.^27^

### Experimental model and study participant details

This study was reviewed by the Mayo Clinic Institutional Review Board as a minimal-risk study and determined to be exempt. Participants were excluded if they did not have a research authorization on file. Further information on the Mayo Clinic Institutional Review Board and adherence to basic ethical principles underlying the conduct of research and ensuring that rights and well-being of potential research participants are adequately protected are available at the Mayo Clinic website.

#### Study design

In the de-identified EHR database, the study population included all individuals with at least one positive SARS-CoV-2 PCR test between June 1, 2021 (four months before the first use of the long COVID ICD-10 code) to May 28, 2022. Individuals without a primary care provider on record in the health system or with no clinical encounters recorded in the past three years were excluded from the analysis. Individuals with at least one ICD-10 code for long COVID (U09.9, “Post COVID-19 condition, unspecified”) at least 7 days after a positive SARS-CoV-2 PCR test were classified as “Long COVID” patients, and the rest of the study population without this ICD-10 code were classified as possible control patients For the long COVID patients, the date of the most recent positive PCR test prior to the first U09.9 ICD-10 code was considered to be the index date. For control patients the date of the first positive PCR test during the study period was considered to be the index date. In Figure 1, we provide an overview of the study design. Information on sex, ancestry/race and ethnicity, and age of all participants is provided in Table S1 (all participants) and Table S4 (matched cohorts).

#### Definition of the matched cohort of control patients

To identify risk factors for long COVID, we constructed a 1:1 matched control cohort starting from the unmatched study population. This cohort was exactly matched on potentially confounding factors for long COVID ICD-10 diagnosis, including demographics (age, sex, race, ethnicity), health system site, date of infection (within two weeks), and the number of lab test encounters at the health system within the past year. Individuals in the long COVID cohort without a corresponding matched control (54 out of 1,140 individuals) were dropped from the matched analysis (see Table S1 for a comparison of the unmatched populations).

### Method details

#### Extraction of phenotypes from clinical notes

A Bidirectional Encoder Representations from Transformers (BERT)-based classification model (Methods S1) was used to classify the sentiment for phenotypes, defined as symptoms and health conditions, mentioned in EHR clinical notes. BERT is a transformer-based machine learning model used for natural language processing of unlabeled data. This model was previously used to identify signs and symptoms of COVID-19,^28^ short and long-term complications of COVID-19,^29^ and adverse events of mRNA-based COVID-19 vaccines.^30^

For this study, we applied the BERT model to classify the sentiment of 64 phenotypes (Table S2) in the clinical notes for individuals in the long COVID and control groups during each of the study phases. This list of phenotypes was obtained from the CDC website for long COVID^13^ and publicly available literature sources, and the methodology to identify candidate long COVID phenotypes from publicly available literature sources is described in the following STAR Methods section. It is important to note that this is not an exhaustive list of all COVID-19 phenotypes, but a starting point for characterizing long COVID-10. Also, it is important to note that the BERT model allows patients to be associated with multiple phenotypes. This means that each patient’s notes are considered for all phenotypes of interest, enabling a comprehensive analysis of potential associations.

For the analysis of clinical notes, we first define the following phases: the baseline or pre-COVID-19 phase (10–365 days before infection), the acute COVID-19 phase (0–14 days after infection), and the post-COVID-19 phase (28–42 days after infection). The time window for the post-COVID-19 phase was selected to both capture most new long COVID diagnosis (60% of the long COVID cohort was diagnosed with long COVID before day 42, and 18% was diagnosed between day 28–42) and because during this time window we observed significant differences in overall phenotype reporting (Figure S1). Individuals without at least one clinical note during the baseline phase and individuals with less than 42 days of follow-up post-PCR were excluded from this analysis.

#### Identification of candidate long COVID phenotypes from publicly available literature sources

Using the nferX Signals Application, we identified disease phenotypes frequently mentioned in the biomedical literature in the context of long COVID and its associated synonyms (e.g., “pasc”, “post-COVID condition”). The full list of disease phenotypes that were considered includes approximately 140K unique phenotypes compiled from 9 sources which are available in the nferX “Diseases” collection (see Table S3). The synonym lists for each of these phenotypes and “long COVID” were determined by the nferX Signals application. For each phenotype in the “Diseases” collection, we computed a metric called the “nferX local score” (Methods S1) which measures the strength of the association between that phenotype and long COVID in the nferX corpus of biomedical literature. The final list of 64 phenotypes considered for this study is the union of the list of phenotypes on the CDC website for long COVID^13^ and the list of candidate long COVID phenotypes identified by the nferX Signals application (see Table S2).

### Quantification and statistical analysis

#### Comparison of lab measurements

For the matched long COVID and control groups, we computed: (a) the mean values of a lab test for each patient contributing to the analysis of this lab test (mean_individual_: patient-level data summarization), and (b) the mean values of a lab test for all mean_individual_ values of patients in a cohort (mean_population_: population-level data summarization). We performed these calculations for the baseline and acute COVID-19 phases. We compared the mean_population_ (referred to as ‘mean’ in the supplementary tables) values for a lab test between — i) the baseline and acute COVID-19 phases for the long COVID cohort, and ii) the long COVID and control groups in the acute COVID-19 phase. In both cases, we report *p*-values from Mann-Whitney U tests, subsequently corrected for multiple comparisons using the Benjamini-Yekutieli (False Discovery Rate) method. We also calculated 95% confidence intervals around the mean_population_ values by bootstrap resampling (1000 samples).

#### Comparison of clinical characteristics

We compared the clinical characteristics of the long COVID and control groups and reported odds ratios and 95% confidence intervals. For age, we considered the following buckets: <18, 18–24, 25–34, 35–44, 45–54, 55–64, 65–74, and 75+ years old. For race, we grouped the categories (“Asian,” “Asian - Far East,” and “Asian - Indian Subcontinent”) as “Asian,” and we grouped the categories (“Chose not to disclose,” “Unable to provide,” and “Unknown”) as “Unknown.” For ethnicity, we grouped the categories (“Choose not to disclose” and “Unknown”) as “Unknown.” For the number of lab test encounters within the past year, we considered the following buckets: 0, 1–3, and 4+ lab test encounters. Individuals with at least one dose of the Janssen COVID-19 vaccine or two or more doses of the Pfizer or Moderna COVID-19 vaccines on record were considered to be fully vaccinated. Comorbidities were determined based on ICD codes observed during the baseline phase. Comorbidities in the Charlson Comorbidity Index^31^ were considered along with auto-immune diseases and related conditions, including chronic fatigue syndrome, postural tachycardia syndrome without hypotension, fibromyalgia, and migraine. We also compared medications administered or ordered for the matched long COVID and control groups during the baseline, acute COVID-19, and post-COVID-19 phases. We report *p*-values from Fisher’s exact test performed for each phase.

## Supplemental information

Document S1. Figures S1–S6 and Tables S1–S4 and S6 and Method S1

Table S5. List of lab tests in the acute COVID-19 phase, related to Figure 1

## References

[1] Clarke K.E.N., Jones J.M., Deng Y., Nycz E., Lee A., Iachan R., Gundlapalli A.V., Hall A.J., MacNeil A. (2022). Seroprevalence of Infection-Induced SARS-CoV-2 Antibodies - United States, September 2021-February 2022. MMWR Morb. Mortal. Wkly. Rep..

[2] CDC (2020). COVID Data Tracker. Centers for Disease Control and Prevention. https://covid.cdc.gov/covid-data-tracker.

[3] Tan B.K.J., Han R., Zhao J.J., Tan N.K.W., Quah E.S.H., Tan C.J.-W., Chan Y.H., Teo N.W.Y., Charn T.C., See A. (2022). Prognosis and persistence of smell and taste dysfunction in patients with covid-19: meta-analysis with parametric cure modelling of recovery curves. BMJ.

[4] (2021). A clinical case definition of post COVID-19 condition by a Delphi consensus, 6 October 2021. https://www.who.int/publications/i/item/WHO-2019-nCoV-Post_COVID-19_condition-Clinical_case_definition-2021.1.

[5] (2022). Long COVID. https://www.cdc.gov/nchs/covid19/pulse/long-covid.htm#technical_notes.

[6] Subramanian A., Nirantharakumar K., Hughes S., Myles P., Williams T., Gokhale K.M., Taverner T., Chandan J.S., Brown K., Simms-Williams N. (2022). Symptoms and risk factors for long COVID in non-hospitalized adults. Nat. Med..

[7] O’Laughlin K.N., Thompson M., Hota B., Gottlieb M., Plumb I.D., Chang A.M., Wisk L.E., Hall A.J., Wang R.C., Spatz E.S. (2022). Study protocol for the Innovative Support for Patients with SARS-COV-2 Infections Registry (INSPIRE): A longitudinal study of the medium and long-term sequelae of SARS-CoV-2 infection. PLoS One.

[8] RECOVER (2022). Researching COVID to Enhance Recovery RECOVER: Researching COVID to Enhance Recovery.

[9] Wagner T., Shweta F.N.U., Murugadoss K., Awasthi S., Venkatakrishnan A.J., Bade S., Puranik A., Kang M., Pickering B.W., O’Horo J.C. (2020). Augmented curation of clinical notes from a massive EHR system reveals symptoms of impending COVID-19 diagnosis. Elife.

[10] Venkatakrishnan A.J., Pawlowski C., Zemmour D., Hughes T., Anand A., Berner G., Kayal N., Puranik A., Conrad I., Bade S. (2021). Mapping each pre-existing condition’s association to short-term and long-term COVID-19 complications. NPJ Digit. Med..

[11] McMurry R., Lenehan P., Awasthi S., Silvert E., Puranik A., Pawlowski C., Venkatakrishnan A.J., Anand P., Agarwal V., O’Horo J.C. (2021). Real-time analysis of a mass vaccination effort confirms the safety of FDA-authorized mRNA COVID-19 vaccines. Med (N Y).

[12] Charlson M.E., Pompei P., Ales K.L., MacKenzie C.R. (1987). A new method of classifying prognostic comorbidity in longitudinal studies: development and validation. J. Chronic Dis..

[13] Chen C., Zhang Y., Zhao X., Tao M., Yan W., Fu Y. (2021). Hypoalbuminemia – An Indicator of the Severity and Prognosis of COVID-19 Patients: A Multicentre Retrospective Analysis. Infect. Drug Resist..

[14] Lim S., Bae J.H., Kwon H.-S., Nauck M.A. (2021). COVID-19 and diabetes mellitus: from pathophysiology to clinical management. Nat. Rev. Endocrinol..

[15] Montefusco L., Ben Nasr M., D’Addio F., Loretelli C., Rossi A., Pastore I., Daniele G., Abdelsalam A., Maestroni A., Dell’Acqua M. (2021). Acute and long-term disruption of glycometabolic control after SARS-CoV-2 infection. Nat. Metab..

[16] Barrett C.E., Koyama A.K., Alvarez P., Chow W., Lundeen E.A., Perrine C.G., Pavkov M.E., Rolka D.B., Wiltz J.L., Bull-Otterson L. (2022). Risk for Newly Diagnosed Diabetes 30 Days After SARS-CoV-2 Infection Among Persons Aged 18 Years — United States, March 1, 2020–June 28, 2021. MMWR Morb. Mortal. Wkly. Rep..

[17] Scherer P.E., Kirwan J.P., Rosen C.J. (2022). Post-acute sequelae of COVID-19: A metabolic perspective. Elife.

[18] Reiterer M., Rajan M., Gómez-Banoy N., Lau J.D., Gomez-Escobar L.G., Ma L., Gilani A., Alvarez-Mulett S., Sholle E.T., Chandar V. (2021). Hyperglycemia in acute COVID-19 is characterized by insulin resistance and adipose tissue infectivity by SARS-CoV-2. Cell Metab..

[19] Martínez-Colón G.J., Ratnasiri K., Chen H., Jiang S., Zanley E., Rustagi A., Verma R., Chen H., Andrews J.R., Mertz K.D. (2022). SARS-CoV-2 infection drives an inflammatory response in human adipose tissue through infection of adipocytes and macrophages. Sci. Transl. Med..

[20] CDC (2022). Public Health Recommendations. Centers for Disease Control and Prevention. https://www.cdc.gov/coronavirus/2019-ncov/hcp/clinical-care/post-covid-public-health-recs.html.

[21] Groenwold R.H.H. (2020). Informative missingness in electronic health record systems: the curse of knowing. Diagn. Progn. Res..

[22] (2020). Tests and procedures. https://www.mayoclinic.org/tests-procedures.

[23] (2022). Test catalog - mayo clinic laboratories. https://www.mayocliniclabs.com/test-catalog.

[24] Diagnostics (2022). Testing Cleveland Clinic. https://my.clevelandclinic.org/health/diagnostics.

[25] (2022). Medical Tests ucsfhealth.org. https://www.ucsfhealth.org/medical-tests.

[26] Murugadoss K., Rajasekharan A., Malin B., Agarwal V., Bade S., Anderson J.R., Ross J.L., Faubion W.A., Halamka J.D., Soundararajan V., Ardhanari S. (2021). Building a best-in-class automated de-identification tool for electronic health records through ensemble learning. Patterns (N Y).

[27] CDC (2022). Long COVID or Post-COVID Conditions. Centers for Disease Control and Prevention. https://www.cdc.gov/coronavirus/2019-ncov/long-term-effects/index.html.

[28] Pfaff E.R., Girvin A.T., Bennett T.D., Bhatia A., Brooks I.M., Deer R.R., Dekermanjian J.P., Jolley S.E., Kahn M.G., Kostka K. (2022). Identifying who has long COVID in the USA: a machine learning approach using N3C data. Lancet. Digit. Health.

[29] Masana L., Correig E., Ibarretxe D., Anoro E., Arroyo J.A., Jericó C., Guerrero C., Miret M., Näf S., Pardo A. (2021). Low HDL and high triglycerides predict COVID-19 severity. Sci. Rep..

[30] Huang J., Cheng A., Kumar R., Fang Y., Chen G., Zhu Y., Lin S. (2020). Hypoalbuminemia predicts the outcome of COVID-19 independent of age and co-morbidity. J. Med. Virol..

[31] Wagner J., Garcia-Rodriguez V., Yu A., Dutra B., Larson S., Cash B., DuPont A., Farooq A. (2021). Elevated transaminases and hypoalbuminemia in Covid-19 are prognostic factors for disease severity. Sci. Rep..

