# Supplementary material for: Case-control study on post-COVID-19 conditions reveals severe acute infection and chronic pulmonary disease as potential risk factors
Source: iScience. 2024 Jun 28;27(8):110406. doi: 10.1016/j.isci.2024.110406 (PMC11284568; doi:10.1016/j.isci.2024.110406)
Supplement: Document S1. Figures S1–S6 and Tables S1–S4 and S6 and Method S1 [file mmc1.pdf]

iScience, Volume *27*

Supplemental information

Case-control study on post-COVID-19 conditions reveals severe acute infection and chronic pulmonary disease as potential risk factors

Pritha Ghosh, Michiel J.M. Niesen, Colin Pawlowski, Hari Bandi, Unice Yoo, Patrick J. Lenehan, Praveen Kumar-M, Mihika Nadig, Jason Ross, Sankar Ardhanari, John C. O'Horo, A.J. Venkatakrishnan, Clifford J. Rosen, Amalio Telenti, Ryan T. Hurt, and Venky Soundararajan

Included material:

Methods S1: Additional details on previously developed methods used, related to STAR methods.

Figure S1: Reporting of any tracked phenotype as a function of time, related to Figure 1.
Figure S2: Subtypes of chronic pulmonary disorder in the long COVID and control patients, related to Table 1.
Figure S3: Lab test enrichments for matched long COVID and control groups, related to Figure 1c.
Figure S4: Lab test enrichments for matched long COVID and control patients, related to Figure 1c.
Figure S5: Comparison of medications administered or ordered for matched long COVID and control patients, related to main text results on medication use.
Figure S6: Comparison of medications administered or ordered for matched long COVID and control patients for the baseline phase, related to main text results on medication use.

Table S1: Clinical characteristics, comorbidities, and clinical outcomes of long COVID and pre-matching control population, related to STAR methods.
Table S2: List of long COVID phenotypes identified by CDC and nferX Signals, related to STAR methods and main text Figure 1.
Table S3: List of data sources for nferX Diseases collection, related to STAR methods.
Table S4: Clinical characteristics of long COVID and matched control patients, related to STAR methods.
Table S6: Comparison of medications administered or ordered during the acute, post COVID-19 and baseline phases, related to main text results on medication use.

Methods S1: Additional details on previously developed methods used, related to STAR methods.

nferX Signals Platform

The nferX Signals application (https://research.nferx.com/dv/202011/signals/) was used to determine candidate long COVID phenotypes from publicly available literature sources. This application enables the user to search for biomedical associations in free-text over 100 million documents from over 80K sources including but not limited to: PubMed articles, clinicaltrials.gov, patent applications, SEC filings, blogs, conferences, and news articles.

nferX Local Score

The nferX Local Score is the metric that the nferX Signals Platform uses to assess the association between two biomedical concepts in the literature. The local score measures how frequently two tokens are found within each other's local context in a particular corpus, normalized by the occurrences of those tokens in that corpus. We define the local context of a particular token as the five tokens immediately preceding and following every occurrence of that token. We additionally define the adjacency adj_{AB}between tokens A and B as the number of items token A is found in token B's local context, or vice-versa. We calculate the pointwise mutual information pmi_{AB}between tokens A and B as the following:

$$pmi_{AB} = log_{10}(\frac{ajd_{AB} \cdot N_c}{N_A \cdot N_B})$$

Where $N_A$is the occurrences of token A, $N_B$is the occurrences of token B, and N_C is the summed occurrences of all tokens in the corpus of interest. We then calculate the local score LS_{AB} between tokens A and B as the following:

$$LS_{AB} = ln(adj_{AB} + 1) \cdot \frac{1}{1 + e^{-(pmi_{AB} - 1.5)}}$$

For this study, we used the nferX Signals application to compute local scores between "long COVID" and ~80K potential disease phenotypes from the nferX "Diseases" collection. The disease phenotypes with the highest local scores (and therefore highest literature associations to "long COVID") are shown in Table S2. The sources for the nferX "Diseases" collection are described in Table S3.

Phenotype Classification Using BERT

A Bidirectional Encoder Representations from Transformers (BERT)-based classification model was used to classify the sentiment for phenotypes, defined as symptoms and health conditions, mentioned in EHR clinical notes. Given a sentence that includes any phenotype, this model outputs one of the following labels: "Yes" - confirmed diagnosis, "Maybe" - possible diagnosis, "No" - ruled out the diagnosis, or "Other" - none of the above. A dataset of 18,490 manually annotated sentences extracted from EHR clinical notes containing over 250 different phenotypes was used to train the model. The classification model achieves an out-of-sample accuracy of 93.6% and precision and recall values above 95%.[10]

In this study and consistent with previously published studies, we used the following criteria for counting an individual as positive for a phenotype. For the baseline phase, an individual was counted as positive for the phenotype if they had at least one mention of the phenotype with a “Yes” label and the confidence score was greater than 0.8 (a “positive sentiment”). For each prediction, the confidence score is a number between 0 and 1 which reflects the certainty of the model that the prediction is correct, with 0 being the least certain and 1 being the most certain. We selected a threshold of 0.8 for the confidence score consistent with prior studies and validated using manual review of a subset of model predictions. For the acute and post-COVID-19 phases, an individual was counted as positive for a phenotype only if they had a positive sentiment for the phenotype during that phase (i.e. “Yes” label and confidence score > 0.8) without any positive sentiment in the baseline phase. We term such phenotypes as “new onset”. We have also quantified the overall prevalence of positive sentiments for any of the 64 phenotypes during 7-day intervals from 42 days before the positive PCR test to 42 days after the positive PCR test (Figure S1).

Supplementary Figures

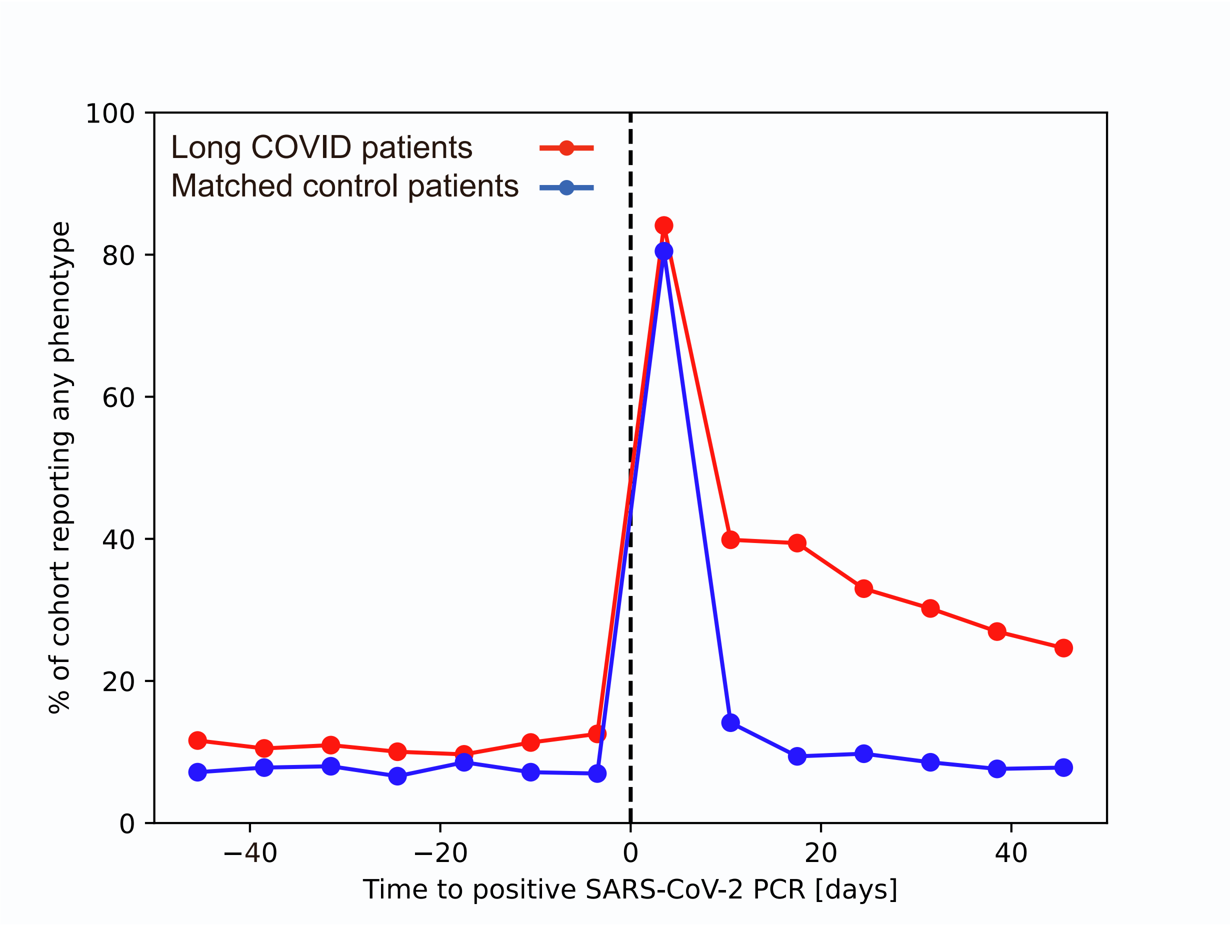

Figure S1: Reporting of any tracked phenotype as a function of time, related to Figure 1. Data shown for the long COVID cohort (red) and their 1:1 matched controls (blue). The vertical dashed line indicates the date of the patient's positive SARS-CoV-2 PCR test.

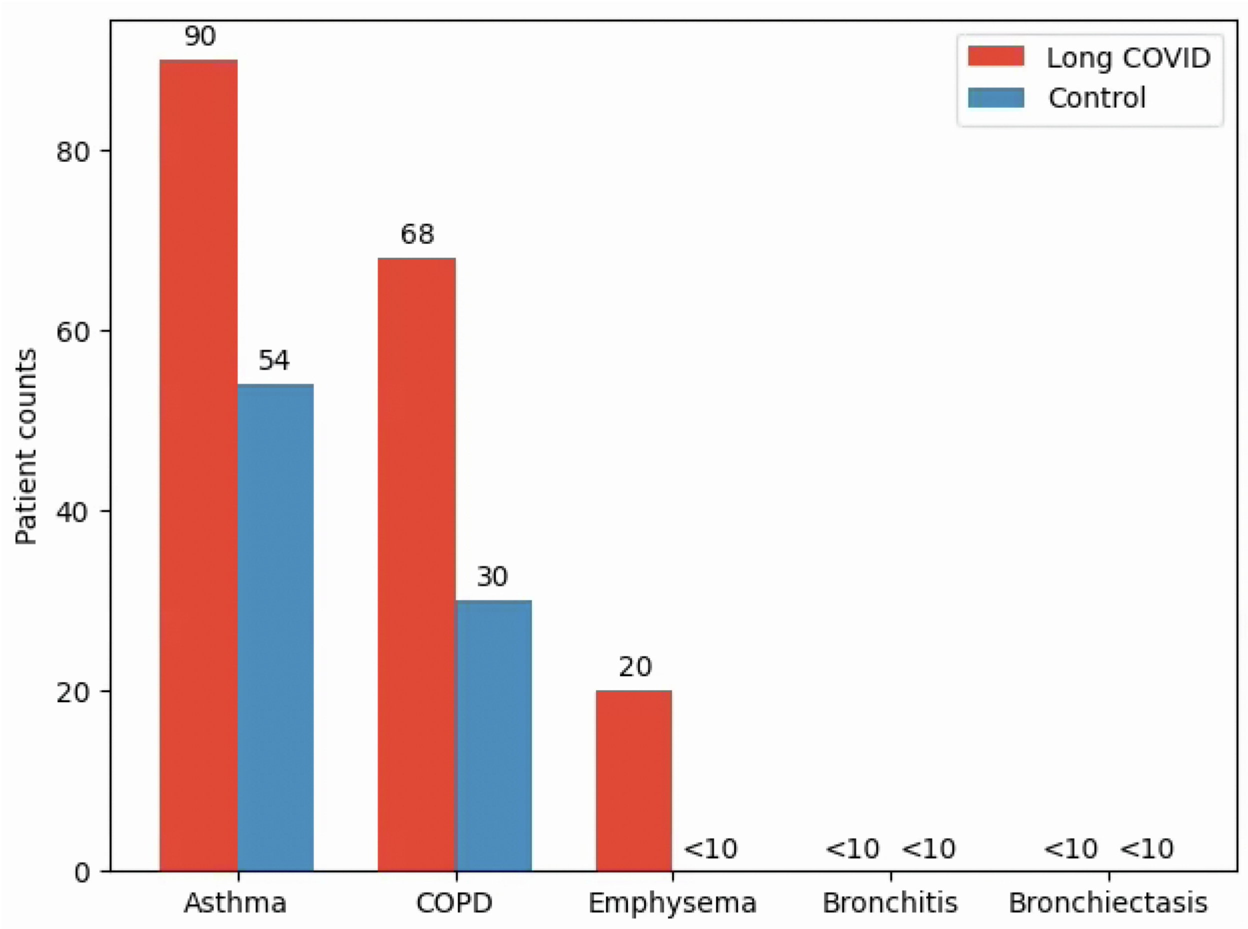

Figure S2: Subtypes of chronic pulmonary disorder in the long COVID and control patients, related to Table 1.

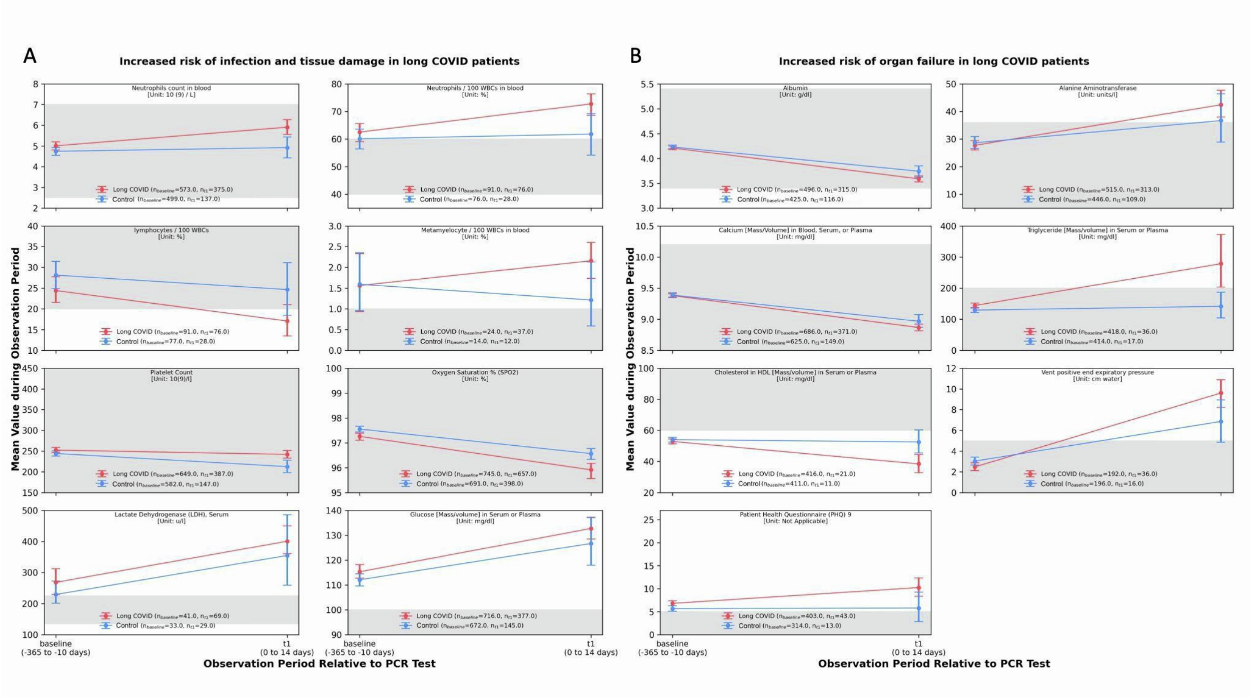

Figure S3: Lab test enrichments for matched long COVID and control groups, related to Figure 1c. For each lab test, mean test values for the long COVID cohort were compared to those of the control group (see *Methods*). The error bars represent 95% confidence intervals, calculated by bootstrap resampling (1000 samples). The normal ranges for these lab tests[28–31] are shaded in gray. Fifteen lab tests shown here are significantly different (Mann Whitney U test, p-value < 0.05) between the long COVID and the control patients in the acute COVID-19 phase and also significantly different between the long COVID cohorts in the baseline and acute COVID-19 phases. **(a)** Lab tests indicating infection and tissue damage **(b)** Lab tests indicating risk of organ failure.

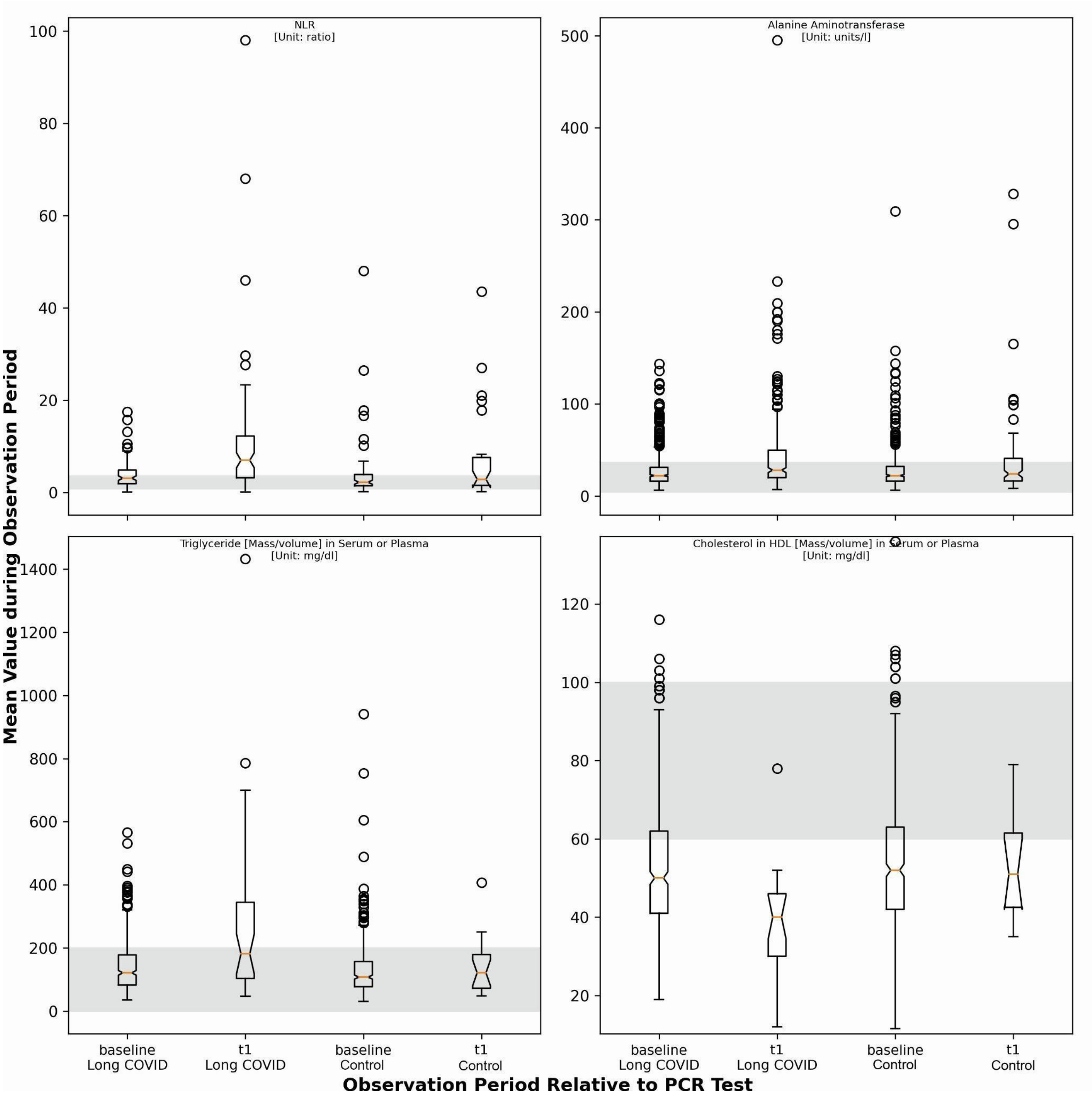

Figure S4: Lab test enrichments for matched long COVID and control patients, related to Figure 1c. For each lab test, the distribution of $mean_{individual}$ test values for the long COVID cohort were compared to the control patients (see *Methods*). The error bars represent 95% confidence intervals, calculated by bootstrap resampling (1000 samples). The normal ranges for these lab tests[28–31] are shaded in gray. Here we show four of these 15 lab tests significantly enriched in the long COVID cohort (see *Methods*) with mean test values outside the normal range.

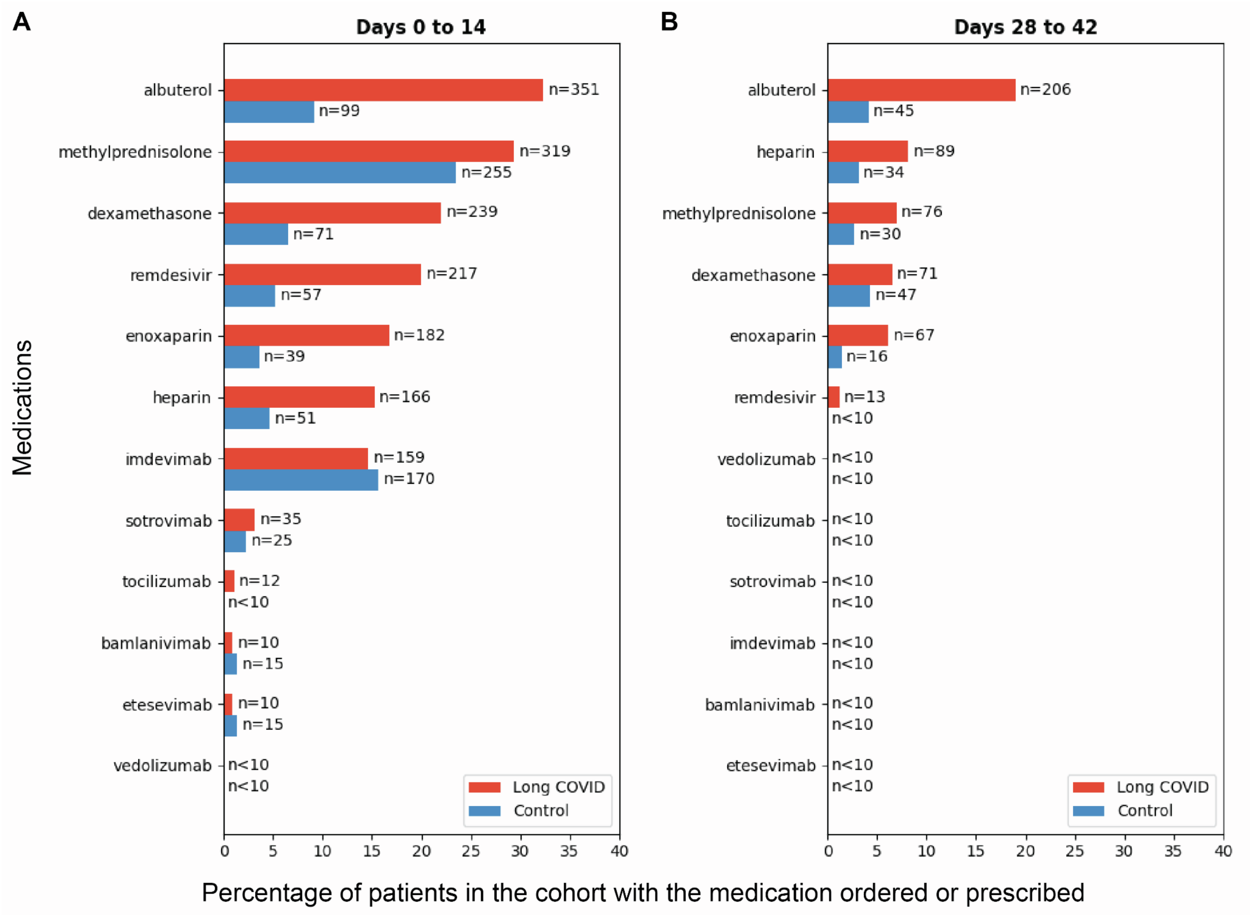

Figure S5: Comparison of medications administered or ordered for matched long COVID and control patients, related to main text results on medication use. Medications administered or ordered during the acute COVID-19 phase, **(A)**, the post-COVID-19 phase, **(B)** for the matched long COVID and control patients.

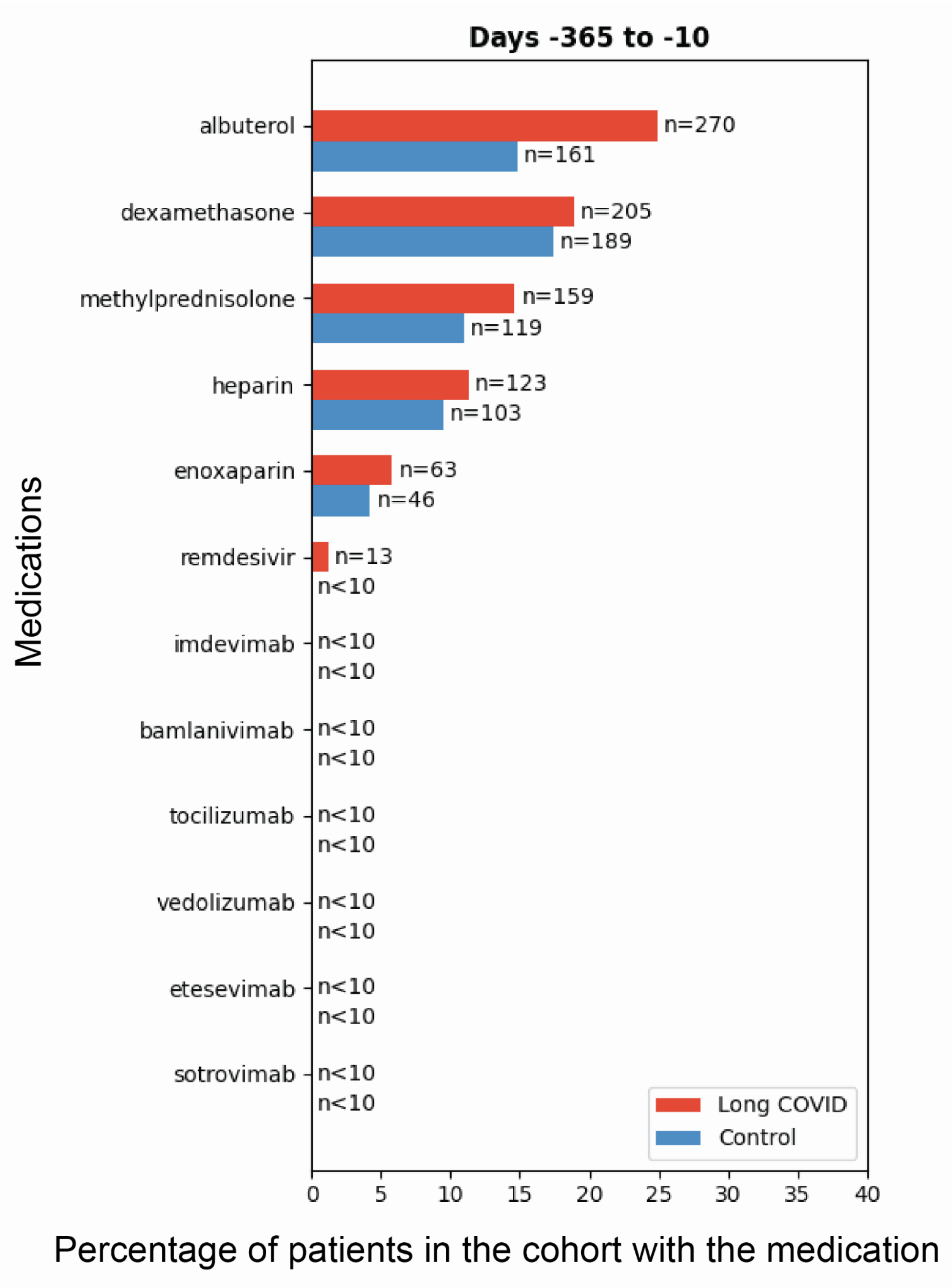

Figure S6: Comparison of medications administered or ordered for matched long COVID and control patients for the baseline phase, related to main text results on medication use.

Table S1: Clinical characteristics, comorbidities, and clinical outcomes of long COVID and pre-matching control population, related to STAR methods. For each categorical variable, the percentage of patients in each cohort is shown along with the odds ratio and corresponding 95% confidence interval. Odds ratios that are statistically significant (p-value < 0.05) are indicated with *, and those that are highly significant (p-value < 0.001) are indicated with ***.

	Long COVID cohort	Unmatched control population	Odds Ratio [95% CI]
**Number of individuals**	1,140	87,803	-
**Age at COVID diagnosis**			
- Median age (IQR)	54 (40, 67)	39 (21, 58)	-
**Patient counts (%)**			
- <18 years old	4	21	0.15 [0.11, 0.21]***
- 18-24 years old	4	8	0.43 [0.32, 0.59]***
- 25-34 years old	10	14	0.65 [0.53, 0.79]***
- 35-44 years old	16	15	1.06 [0.9, 1.25]
- 45-54 years old	18	13	1.53 [1.31, 1.78]***
- 55-64 years old	20	13	1.68 [1.45, 1.94]***
- 65-74 years old	16	10	1.74 [1.48, 2.04]***
- ≥ 75 years old	13	7	2.14 [1.8, 2.54]***
**Sex (%)**			
- Female	62	53	1.43 [1.26, 1.61]***
- Male	38	47	0.7 [0.62, 0.79]***
**Race (%)**			
- White	91	89	1.27 [1.04, 1.56]*
- Black/African American	3	4	0.72 [0.51, 1.02]
- Native American / Pacific Islander	<1	<1	-
- Asian	3	3	5.12 [4.28, 6.11]***
- Other	1	3	0.47 [0.28, 0.79]*
- Unknown	1	1	1.1 [0.67, 1.8]
**Ethnicity (%)**			
- Not Hispanic or Latino	93	92	1.14 [0.91, 1.42]
- Hispanic or Latino	6	7	0.87 [0.68, 1.12]
- Unknown	1	1	0.95 [0.6, 1.49]
**Geographic site (%)**			
- MCHS	55	59	0.85 [0.76, 0.96]*
- MN	17	21	0.78 [0.67, 0.91]***
- AZ	16	10	1.7 [1.45, 1.99]***
- FL	11	9	1.21 [1.0, 1.45]*
**Number of encounters (%)**			
- 0	10	19	0.48 [0.39, 0.58]***
- 1-3	34	47	0.58 [0.51, 0.66]***
- 4+	56	34	2.46 [2.19, 2.77]***
**Fully vaccinated before infection[1] (%)**			
- Pfizer (two or more doses)	37	35	1.08 [0.95, 1.22]
- Moderna (two or more doses)	16	15	1.13 [0.96, 1.32]
- Janssen (one or more doses)	5	4	1.42 [1.09, 1.85]*

	Long COVID cohort	Unmatched control population	Odds Ratio [95% CI]
- Any other vaccine (two or more doses)	0	<1	-
**Comorbidities in baseline phase (%)**			
- Cancer	6	3	1.96 [1.53, 2.52]***
- Cerebrovascular disease	3	2	1.76 [1.24, 2.48]*
- Chronic pulmonary disease	15	6	2.65 [2.24, 3.13]***
- Congestive heart failure	8	3	2.57 [2.06, 3.21]***
- Dementia	<1	<1	-
- Diabetes without chronic complication	12	6	2.09 [1.74, 2.51]***
- Hemiplegia or paraplegia	<1	<1	-
- Metastatic solid tumor	1	<1	1.56 [0.96, 2.53]
- Mild liver disease	4	2	1.67 [1.22, 2.28]*
- Moderate or severe liver disease	<1	<1	-
- Myocardial infarction	3	1	2.32 [1.60, 3.37]***
- Peptic ulcer disease	1	<1	2.55 [1.52, 4.29]***
- Peripheral vascular disease	7	3	2.28 [1.81, 2.87]***
- Renal disease	13	5	2.81 [2.36, 3.34]***
- Rheumatic disease	5	2	3.04 [2.32, 3.99]***
- at least one of the listed comorbidities	40	20	2.54 [2.25, 2.86]***
**Auto-immune diseases and potentially related conditions in baseline phase (%)**			
- Chronic Fatigue Syndrome	1	<1	4.69 [2.78, 7.92]***
- Postural Tachycardia Syndrome Without Hypotension	<1	<1	-
- Fibromyalgia	4	1	3.93 [2.9, 5.34]***
- Migraine	10	4	2.52 [2.06, 3.07]***
- at least one of the listed conditions	13	5	2.83 [2.37, 3.37]***
**Individuals admitted 0-14 days post-infection (%)**			
- Hospitalized	6	<1	9.77 [7.5, 12.73]***
- ICU admission	3	<1	11.52 [8.0, 16.59]***
- Intubated	3	<1	4.63 [3.34, 6.44]***

Table S2: List of long COVID phenotypes identified by CDC and nferX Signals, related to STAR methods and main text Figure 1. In the first two columns, the phenotype names are shown along with the data source (e.g. CDC, Signals, or both). In the third column, the nferX Local Score is shown, which is a measure of the strength of the association between that phenotype and long COVID in the biomedical literature. Phenotypes with the highest local score values are most strongly associated with long COVID in the literature.

Phenotype	Source	nferX Local Score
brain_fog	CDC and Signals	6.972
anosmia	Signals	5.155
muscle_pain	CDC and Signals	5.128
severe_covid_19_disease	Signals	5.121
fibromyalgia	Signals	5.054
encephalomyelitis	Signals	4.89
chest_pain	CDC and Signals	4.746
dysautonomia	Signals	4.729
post_exertional_malaise	CDC and Signals	4.683
headache	CDC and Signals	4.551
acute_respiratory_syndrome	Signals	4.531
neurologic_manifestations	Signals	4.527
cognitive_dysfunction	Signals	4.508
cough	CDC and Signals	4.372
joint_pain	CDC and Signals	4.323
post_intensive_care_syndrome	CDC and Signals	4.299
orthostatic_intolerance	Signals	4.257
post_concussion_syndrome	Signals	4.238
pancreatic_adenosquamous_carcinoma	Signals	4.199
severe_acute_respiratory_syndrome	Signals	4.122
solid_tumor	Signals	4.087
anxiety	CDC and Signals	4.059
ageusia	Signals	4.05
myocarditis	Signals	3.998
dysgeusia	CDC and Signals	3.959
adenosquamous_carcinoma	Signals	3.945
pacs	Signals	3.911
cocaine_intoxication	Signals	3.897
pulmonary_fibrosis	Signals	3.827
muscle_weakness	CDC and Signals	3.741
tiredness	CDC and Signals	3.634
immune_dysregulation	Signals	3.615
parosmia	CDC and Signals	3.601
postural_orthostatic_tachycardia_syndrome	Signals	3.599
pancreatic_ductal_adenocarcinoma	Signals	3.56
depression	CDC and Signals	3.548
mast_cell_activation_syndrome	Signals	3.521
non_alcoholic_steatohepatitis	Signals	3.479

alcoholic_steatohepatitis	Signals	3.415
body_aches	Signals	3.395
viral_infection	Signals	3.366
post_infectious_syndromes	Signals	3.315
inappropriate_sinus_tachycardia	Signals	3.298
fever	CDC and Signals	3.189
poisoning	Signals	3.181
cognitive_deficits	Signals	3.179
acute_respiratory_distress_syndrome	Signals	3.178
lung_disease	Signals	3.127
hepatic_encephalopathy	Signals	3.075
secondary_lymphedema	Signals	3.009
autoimmune_conditions	CDC	< 3
changes_in_menstrual_cycles	CDC	< 3
diabete_mellitus	CDC	< 3
diarrhea	CDC	< 3
difficulty_breathing	CDC	< 3
heart_palpitations	CDC	< 3
lightheadedness	CDC	< 3
multisystem_inflammatory_syndrome	CDC	< 3
myalgic_encephalomyelitis	CDC	< 3
pins_and_needles_feelings	CDC	< 3
post_traumatic_stress_disorder	CDC	< 3
rash	CDC	< 3
sleep_problems	CDC	< 3
stomach_pain	CDC	< 3

Table S3: List of data sources for nferX Diseases collection, related to STAR methods.

Source	Url	Description
GARD	https://www.gard.no/web/frontpage	GARD provides the public with access to current, reliable, and easy to understand information about rare or genetic diseases in English or Spanish. Who can GARD help with information? People who have rare or genetic diseases. Parents, family members, and friends of someone with a rare or genetic disease.
MEDLINE	https://www.medline.com/	MEDLINE is a bibliographic database of life sciences and biomedical information. It includes bibliographic information for articles from academic journals covering medicine, nursing, pharmacy, dentistry, veterinary medicine, and health care.
NORD	https://rarediseases.org/	The National Organization for Rare Disorders (NORD) maintains a database that includes reports on over 1,200 rare diseases.
MONDO_DISEASE_ONTOLOGY	https://www.ebi.ac.uk/ols/ontologies/mondo	A semi-automatically constructed ontology that merges in multiple disease resources to yield a coherent merged ontology.
MAYO	https://www.mayoclinic.org/	The Mayo Clinic is a nonprofit American academic medical center focused on integrated health care, education, and research.
UNIPROT_DISEASE	https://www.uniprot.org/keywords/9995	The Universal Protein Resource (UniProt) is a comprehensive resource for protein sequence and annotation data. The UniProt databases are the UniProt Knowledgebase (UniProtKB), the UniProt Reference Clusters (UniRef), and the UniProt Archive (UniParc). The UniProt consortium and host institutions EMBL-EBI, SIB and PIR are committed to the long-term preservation of the UniProt databases.
DOID	https://disease-ontology.org/	Disease Ontology is a standardized ontology for human disease maintained by the University of Maryland School of Medicine, Institute for Genome Sciences.
ORPHANET	https://www.orpha.net/consor/cgi-bin/index.php	Orphanet is a European website providing information about rare diseases as well as corresponding diagnosis, orphan drugs, clinical trials and expert networks The website is managed by a consortium of academic establishments from 40 countries, led by Inserm. It contains content both for physicians and for patients.
MESH	https://www.ncbi.nlm.nih.gov/mesh/	Medical Subject Headings is a comprehensive controlled vocabulary for the purpose of indexing journal articles and books in the life sciences. It serves as a thesaurus that facilitates searching.

Table S4: Clinical characteristics of long COVID and matched control patients, related to STAR methods. For each categorical variable, the percentage of patients in each cohort is shown. During the matching procedure, each of the categorical variables were matched exactly, so the distributions are exactly the same for the two cohorts. The numeric variables (age and number of encounters) were bucket matched, so there may be slight differences in these covariates between the two cohorts.

	**Long COVID cohort (matched)**	**Control patients (matched)**
**Number of individuals**	1,086	1,086
**Age at COVID diagnosis (in years)**		
- Median age (IQR)	54 (41 - 68)	54 (40 - 67)
**Age at COVID diagnosis (distribution)**		
- <18 years old	4	4
- 18-24 years old	4	4
- 25-34 years old	9	9
- 35-44 years old	16	16
- 45-54 years old	18	18
- 55-64 years old	19	19
- 65-74 years old	16	16
- ≥ 75 years old	14	14
**Sex (%)**		
- Female	63	63
- Male	37	37
**Race (%)**		
- White	94	94
- Black/African American	3	3
- Native American / Pacific Islander	<1	<1
- Asian	2	2
- Other	<1	1
- Unknown	<1	0
**Ethnicity (%)**		
- Not Hispanic or Latino	95	95
- Hispanic or Latino	5	5
- Unknown	<1	<1
**Geographic site (%)**		
- **MCHS**	57	57
- MN	17	17
- AZ	16	16
- FL	11	11
**Number of encounters (%)**		
- 0	10	10
- 1-3	34	34
- 4+	57	57

Table S6: Comparison of medications administered or ordered during the acute, post COVID-19 and baseline phases, related to main text results on medication use. For each drug, the number of patients in each cohort is shown along with the odds ratio and corresponding 95% confidence interval. Odds ratios that are statistically significant (p-value < 0.05) are indicated with *, and those that are highly significant (p-value < 0.001) are indicated with ***.

Drug	Phase	Long COVID	Control	Odds ratio
vedolizumab	Acute	<10	<10	NA
sotrovimab	Acute	35	25	1.4 [0.84, 2.32]
remdesivir	Acute	217	57	3.81 [2.88, 5.03]***
bamlanivimab	Acute	10	15	0.67 [0.3, 1.48]
etesevimab	Acute	10	15	0.67 [0.3, 1.48]
imdevimab	Acute	159	170	0.94 [0.77, 1.14]
albuterol	Acute	351	99	3.55 [2.88, 4.36]***
tocilizumab	Acute	12	<10	NA
heparin	Acute	166	51	3.25 [2.41, 4.4]***
enoxaparin	Acute	182	39	4.67 [3.34, 6.53]***
acetaminophen	Acute	531	335	1.59 [1.42, 1.77]***
dexamethasone	Acute	239	71	3.37 [2.62, 4.33]***
methylprednisolone	Acute	319	255	1.25 [1.09, 1.44]*
vedolizumab	post-COVID	<10	<10	NA
remdesivir	post-COVID	13	<10	NA
albuterol	post-COVID	206	45	4.58 [3.35, 6.25]***
tocilizumab	post-COVID	<10	<10	NA
heparin	post-COVID	89	34	2.62 [1.78, 3.85]***
enoxaparin	post-COVID	67	16	4.19 [2.44, 7.18]***
acetaminophen	post-COVID	205	101	2.03 [1.62, 2.54]***
dexamethasone	post-COVID	71	47	1.51 [1.06, 2.16]*
methylprednisolone	post-COVID	76	30	2.53 [1.67, 3.83]***
imdevimab	post-COVID	0	0	NA
bamlanivimab	post-COVID	0	0	NA
etesevimab	post-COVID	0	0	NA
sotrovimab	post-COVID	0	0	NA

vedolizumab	Baseline	<10	<10	NA
sotrovimab	Baseline	<10	<10	NA
remdesivir	Baseline	13	<10	NA
bamlanivimab	Baseline	<10	<10	NA
etesevimab	Baseline	<10	<10	NA
imdevimab	Baseline	<10	<10	NA
albuterol	Baseline	270	161	1.68 [1.41, 2.0]***
tocilizumab	Baseline	<10	<10	NA
heparin	Baseline	123	103	1.19 [0.93, 1.53]
enoxaparin	Baseline	63	46	1.37 [0.95, 1.98]
acetaminophen	Baseline	368	319	1.15 [1.02, 1.31]*
dexamethasone	Baseline	205	189	1.08 [0.91, 1.3]
methylprednisolone	Baseline	159	119	1.34 [1.07, 1.67]*
